# Clinique Medicale

**Published:** 1835-07-01

**Authors:** 


### Clinique Medicale, ou Choix d'Observations Recueillies a l'Hôpital de la Charite. Par *G. Andral*, &c. Deuxieme Edition, revue, corrigée, et augmentée par l'Auteur. Tom. V. Maladies de l'Encephale. Londres, Dulau, Soho Square.

[Second Article, continued from our last Number, p. 421.]

Our author having considered in his first book, as we have seen, diseases affecting the membranes of the encephalon, now proceeds to the consideration of those which affect the substance of the brain itself, first commencing with cerebral congestions, or hyperemia.

Before we proceed to the analysis, we must beg leave to make one observation regarding the term hyperemia, as applied to the brain. When an organ is said to be in a state of hyperemia, it is meant that it contains more blood than ordinary; more than it contained in its normal condition. Now we conceive it physically impossible that any change should take place in the absolute quantity of blood circulating in the brain. We see this organ inclosed in an unyielding bony case, by which it is completely protected from the influence of atmospheric pressure, which we know influences the circulation in all other parts of the body, and in fact from all external influence save what is communicated through the blood-vessels which go to it. Owing, therefore, to this the physical condition of the brain, it may be safely asserted that, under ordinary circumstances, there can be no material change in the absolute quantity of blood circulating in it.

The further consideration of this matter would, we apprehend, lead us into that so much disputed, and, as we think, so much misunderstood, question, of the brain's compressibility. In using the term "misunderstood," we must however take care, and not be misunderstood ourselves, and thereby fall into the very error which we are about to deprecate. In saying that the question was misunderstood, we mean that the precise signification of its terms was not agreed on; that the abettors of the brain's compressibility, and those who deny it that property, did not attach precisely the same idea to the term, and were consequently speaking different languages. We have scarcely a doubt, that if both parties compared notes coolly and dispassionately, there would not be found between them that immense quantum discrepat now supposed. We have a no less authority with us than John Locke himself, when we assert that nothing has contributed more to retard the improvement of science and diffusion of knowledge, nothing has tended more to perpetuate disputes among men in all ages and all countries, than the improper use of terms. We shall now present our readers a few of the more interesting cases, and then proceed to the general deduction from them.

CASE 1. *Disease of the Heart of long standing—Sudden Loss of Consciousness and Motion—Speedy Death—Remarkable Injection of the Substance of the Cerebral Hemispheres.*

A woman, 53 years of age, entered the hospital Cochin in the month of March, 1829, presenting the following state: face flushed; lips purple; œdema of the lower extremities; ascites; speech uttered with panting; orthopnœa; pulsations of the heart tumultuous, repelling the ear, perceptible in almost all the parts of the chest, except on the right posteriorly; pulse sunk, contrasting by the smallness of its beats with the strength of those of the heart, regular in other respects, and not frequent. Cough of long standing, dry sonorous râle in different points of the chest; digestive functions duly performed; no perceptible disturbance as far as regards the nervous centres. This woman was considered as affected with hypertrophy of the parietes of the heart with dilatation of the cavities; she was bled, and subjected to the use of digitalis.

Under this treatment, aided by suitable regimen and rest, the dyspnœa and ascites had diminished a little, when one day, on getting up out of bed, she complained of seeing every thing turn round her; she scarcely uttered these words, when she uttered a loud scream, put her hand towards her head, and fell down deprived of consciousness, which she did not recover during the remainder of the day, and died that night.

*Post-mortem.—Cranium.* The arachnoid of the convexity remarkably dry. The grey substance of the circumvolutions has a well-marked rose coloured tint. The medullary substance, which forms in a great measure the nervous mass situated over the ventricles, is traversed by a very great number of red points, each of which constitutes the orifice of a vessel gorged with blood. The ventricles contain very little serum. The optic thalami and corpora striata less injected than the rest of the hemispheres. The sinuses of the dura mater were gorged with liquid black blood. *Thorax.* The lungs were infarcted with an enormous quantity of frothy serum, which flows out in great quantity at every incision made in their parenchyma. Heart very large; its parietes thickened, and its cavities dilated. Its different orifices free; at the base of one of the aortic valves are some slight ossifications, which present no obstruction to the freedom of its action. The aorta presented no other lesion than some cartilaginous and bony patches incrusting its parietes. *Abdomen.* Slate-coloured tint and enlargement of the papillæ of the gastric mucous membrane over a great part of its extent—liver gorged with blood—spleen small and dense.

*Remarks.* Here was a case in which, before the post-mortem, one might have supposed great cerebral hemorrhage had taken place, the patient having presented the symptoms characterising a violent attack of apoplexy. The scream she uttered, her putting her hand towards her head before falling, indicated that she experienced a painful sensation in the brain. Such a cry does not usually precede an attack of apoplexy, it being rather connected with epileptic fits—after once falling without consciousness, she remained deprived of sensation and motion, and at the end of some hours she died in the way that apoplectics generally do. Yet all that was found in the brain was a greater than ordinary injection of the two hemispheres. This certainly is a very slight lesion to account for such violent symptoms. It is probable that a similar injection takes place momentarily in persons who are momentarily seized with giddiness and other signs of cerebral congestion, without any thing fatal resulting from it. This same injection is also no doubt the only lesion in the brain in those cases where all the symptoms of an attack of apoplexy come on, which, after continuing some hours, entirely disappear, without leaving any trace of their existence. It is not probable that, in such circumstances, hemorrhage takes place; for the blood once effused into the cerebral pulp could not be so soon absorbed. We have seen cases of this kind, in which complete hemiplegia, preceded and accompanied by loss of consciousness, and by stertorous respiration, likewise disappeared after the lapse of some hours. In those cases where loss of motion and sensation limited to one side of the body seems to indicate a more deep-seated lesion in the cerebral hemisphere of the opposite side, is it still possible that there may be but mere cerebral hyperemia without any escape of blood from its vessels? The following will prove that such may be the case.

CASE 2. *Attack of Apoplexy supervening on a Chronic Affection of the Thoracic and Abdominal Organs. Hemiplegia. Death two days after this attack. Considerable Injection of the Substance of the Cerebral Hemispheres. No other Lesion in the Nervous Centres.*

A man, 72 years old, entered the Maison Royale de Santé the 7th July, 1830. Six months before he had been operated on for hydrocele. When eighteen years old he had had a copious hemoptysis; more than three glasses full of blood had been expectorated by him in the space of fifteen hours. Since then no return of the hemoptysis—but all his life he was subject to a cough. When we saw him, we discovered, on examining the abdomen, a bosselée tumor, which could be traced into the right hypochondrium, the flank of the same side, to the epigastrium, to level of the umbilicus, and even into the left hypochondrium: this tumor appeared to us to appertain to the liver; it could be pressed without causing pain. For the last two months only, the appetite was lost; the patient had neither thirst, nausea, nor vomiting; the stools were for a long time frequent and of little consistence; the tongue was covered with a thick, somewhat viscid white mucus: there was an evident fluctuation in the abdomen; the legs were œdematous, and the bursæ seemed infiltrated with a considerable quantity of serum. A very loud respiratory murmur without any râle, extending over the entire chest; pulse was frequent; skin hot; a copious deposition of rosacic acid appeared in the urine. During the two following days the tongue became red and dry. On the 10th, at about 3 o'clock in the afternoon, new symptoms suddenly supervene: the patient loses consciousness all at once; and at our visit on the next morning we were told that the preceding night he had an attack of apoplexy: his state then was as follows: he lies on his back; face much injected; eyes closed; on raising the eyelid, we observe the globe of the eye move slowly; on bringing the finger near it, he quickly depresses the eye-brows; the pupils moderately dilated and equally so on both sides; the right commissure of the lips slightly drawn upwards; the left upper extremity, on being raised, falls again by its own weight as an inert mass; no pain evinced on pinching it. The skin of the left lower extremity equally deprived of sensibility, and it seems deprived of all power of motion. On the right, however, the extremities are capable of performing some movements; when the right arm is raised, it is retained in the air by the patient, and does not fall again instantly, as the left. Intelligence entirely gone; the patient resembles a person in a profound sleep; we cannot see his tongue. The pulse lost the frequency it had the preceding days; (bleeding to sixteen ounces—blisters to the legs—purgative lavement.) During the day the patient gave some signs of consciousness, and spoke a little. On the following morning there was a visible improvement—he answered questions with precision—lips and tongue in the natural state—he was also able to perform some motion with the extremities of the left side; but these limbs were evidently weaker than those of the right side: their sensibility was also less—pulse had resumed some frequency. Towards noon all consciousness was again lost; face very much injected, and up to the following morning he remained in a state of coma from which nothing could arouse him. At our visit at 8 o'clock we find him absolutely in the same state as on the preceding evening; respiration stertorous. He died at noon.

*Dissection, 19 Hours after Death.—Cranium.* The meninges very much injected; sinuses of the dura mater full of blood. Through the entire extent of the cerebral hemispheres, every slice of the nervous pulp presented a very remarkable number of red points. In some places these red points, which are the orifices of so many vessels, filled with blood, are so densely collected, that there result from them bright red spots, a franc piece in diameter. *Thorax.* Close adhesions of the left pleura above and behind; the bronchi considerably dilated; at the summit of the left lung there is a black colour, as also an induration of several lobules. In the midst of these lobules, which were become impermeable to air, were found several small bony concretions, all nearly the size of a grain of barley. These concretions are as hard as real bone; several of them were ramified. Beside them were found other concretions, of softer consistence, like lime on which a little water was thrown. Close adhesions of the pleura towards the summit of the right lung; in this summit were discovered cavities communicating with one another, which might at first be taken for caverns, but which a closer examination shewed to be bronchi, very much dilated; around them were several lobules, black and hard; no trace of tubercles; liquid black blood filled the right cavities of the heart; the left ventricle empty; a black clot, of some consistence, distended the left auricle; a little ossification at the base of the aortic valves. Some small bony patches scattered over the aorta. *Abdomen.* Limpid serum in the peritoneum; on the inner surface of the stomach, towards its small curve, was an ulcer, about the breadth of a five-franc piece at least, with everted edges. The tissue constituting its bottom and edges possessed all the characters of encephaloid matter. The liver very voluminous—about two-thirds of it were changed into encephaloid substance: we observed, 1mo, also great development of the circumvolutions of the yellow substance; in several points a bright red colour, seeming to depend on an unnatural development of the vascular tissue: 2do, in other parts was found, mixed with the tissue of the liver, a pale greenish substance, possessing all the characters of fibrine, which had lost a considerable part of its colouring matter, such as is often found in the cavities of the heart: 3tio, on removing still more of its colouring matter, this substance appeared changed into encephaloid matter. Spleen very dense and hard. Between the spleen and kidney was found an encephaloid mass, of the size of a pullet's egg—two other similar masses, the size of a nut, were attached to the great epiploon.

*Remarks.* This case resembles the preceding, in the rapid manner in which the symptoms came on, as well as in the nature of the latter. Here, again, is a group of symptoms, similar in every respect to those characterising an attack of apoplexy; and on opening the body, we find not a trace of hæmorrhage in the brain, but only a very remarkable injection of its vessels. This injection, which gave an almost uniform red tint to some parts of the brain, was equal in both hemispheres; and yet motion was abolished in only one side of the body, precisely as in cases where one of the hemispheres has become the seat of effusion of blood. Another instance of the inadequacy of our present means of investigation, to explain the infinite variety of the symptoms by anatomical lesions: and observe, we cannot here even call the play of sympathies to our aid, for certainly the hemiplegia did not depend on this. The latter symptom disappeared twenty-four hours after it occurred—then it returned, and this circumstance might have inclined one to think, that the cause which produced it was not itself connected with any serious lesion of the brain: the intelligence also returned for a time, but that is also observed in cases of cerebral hemorrhage. The hemoptysis, which appeared in early life and never returned, as also the bony concretions in one of the lungs, the dilatation of the bronchi of the other lung, and the morbid structures seated in several of the abdominal viscera, render this a very remarkable case.

Case 3.—*Signs of Cerebral Congestion existing for several Years.* On a

*sudden, Hemiplegia of the Right Side, not preceded by Loss of Consciousness; subsequently sudden Abolition of Intelligence, Coma, and Death.*

A woman, about 50 years of age, entered the Hôpital Cochin with an ascites of several months' standing. She stated that, for ten years, she had scarcely passed a week without being affected with dizziness so great, as to oblige her to seek support to prevent her from falling. These dizzinesses used to last for some minutes; they were accompanied with tinnitus aurium, and often when they ceased, the patient used to feel a pricking sensation at the ends of the fingers, which were occasionally as if numbed. *There are some days,* she says, *when the objects which I touch are separated from my hand by a piece of velvet.* However she never lost consciousness—intellect clear, and memory good. She expressed a great wish to be tapped, and I yielded to her request. After the fluid was removed, I discovered in the right hypochondrium a large tumor, which extended to the navel, which at this part terminates in a mousse edge, and resembles in every way an enlarged liver. This tumor is found in the epigastrium; it disappears towards the left hypochondrium. Three days after the tapping, the patient became weak, her tongue became a little dry, when, after another attack of dizziness, without loss of consciousness, she felt, as often before, a numbness of both hands, but principally of the right; this numbness continued longer than usual: she fell asleep about eleven at night—on awaking, she could move the extremities of the right side. The following day, complete hemiplegia of the right side, the sensibility of the paralysed limbs still perfect—intellects still good. The two days following the hemiplegia continued. On the third day after the hemiplegia appeared, speech altogether suspended; she can no longer give any sign of intelligence; the four extremities, when raised, fall as inert masses; eyelids closed, and when we raised them, the globes of the eyes remained immoveable. Coma then came, and in two hours after the respiration became stertorous, and she died.

*Post Mortem.—Cranium.* Vessels of the cerebral membranes gorged with blood; remarkable rose-coloured tint of the grey substance of the circumvolutions; unusual injection of the medullary substance of the cerebral hemispheres, equally marked on both sides. *Thorax.* Great infarction of the lungs. Heart sound. *Abdomen.* Occupied by an enormous tumor, which conceals all the other viscera; this was an encysted dropsy of the right ovary—it consisted of two parts, the upper part solid, which, by reason of its situation, form, and relations, had been looked on as a tumor of the liver; the lower one was softer, and gave, on pressure, a manifest sensation of fluctuation. Internally it consisted of a great number of cells, which contained various sorts of fluid.

*Remarks.* Here is another form of cerebral phenomena; and, on examination, the same state of the nervous centres—sanguineous congestion of them, and nothing more. In this case, the patient had been for several years threatened with apoplexy; then, at the end of one of those giddinesses, to which she was subject, she was struck with hemiplegia, and soon after died, amidst the total suspension of the functions of the life of relation: There is this notable difference between the present case and that which precedes it; it is that, in the one, the loss of consciousness coincided with the hemiplegia, whilst, in the case now before us, the paralysis preceded the loss of consciousness. Thus all the combinations of symptoms produced by cerebral hemorrhage may co-exist with a simple sanguineous congestion of the encephalon. In the following case we shall see other symptoms appear, which are no longer those of simple hemorrhage, and are ordinarily regarded as more particularly connected with softening of the brain.

Case 4.—*Pulmonary Phthisis—sudden Loss of Consciousness, with permanent Flexion of the Left upper Extremity—Death, twenty-seven Hours after the Appearance of these Symptoms. No other Lesion in the Nervous Centres, except a bright Red Injection of their Substance.*

A man, 36 years of age, entered the hospital, La Pitié, with all the signs of phthisis, already far advanced. For some days, he complained of a rather acute pain towards the right temple, and a slight numbness of the extremities of the left side; then, on leaving his bed one morning, he fell suddenly, deprived of consciousness. When carried to bed he did not come to himself, and the following morning we found him in the following state. Face very much injected—his attitude that of a person asleep; answers no questions, and appears quite a stranger to every thing passing around him. The left commissure of the lips slightly drawn up. The tongue cannot be seen; the fingers of the left hand strongly flexed on the palm of the same hand, and cannot be extended. The left fore-arm is also flexed on the arm, so as to form with it a very acute angle; the right upper extremity, when raised, falls again as an inert mass, as do also the two lower extremities; pulse small, not frequent; respiration embarrassed. Two hours after we left the patient, the two upper extremities were agitated by convulsive movements, which do not last, and in the afternoon he died.

*Post Mortem.—Cranium.* The substance of the two cerebral hemispheres were very much dotted with numerous red points. *Thorax.* Tubercular excavations in the two lungs. Heart firm, with slight hypertrophy of the parietes of the left ventricle—black, liquid blood in its cavities. *Abdomen.* Greyish tint on the inner surface of the stomach. Numerous tubercles in the small intestine. Liver and kidneys gorged with blood.

*Remarks.* The symptoms here were similar to those which so often announce softening of the brain; and yet there was no trace of such a lesion, and notwithstanding the difference in the phenomena on both sides of the body, a sanguineous congestion, equal on both sides, was all that was discovered. Why was there flexion of the limbs in this case, and not in the preceding cases? Anatomy does not inform us. Is it not a circumstance worthy of remark, that the four cases of cerebral congestion now reported, regarded individuals labouring under chronic affections at the time the brain became congested in them? In three of them, hematosis was for a long time viciated; they were meagre, bloodless, and appeared to be in a condition entirely opposite to that which is usually laid down as favouring cerebral congestions; an additional example to prove, that the facility with which local hyperemias are produced, is not always in the direct ratio of the plethoric state of the subject.

Resumé.

The cases now recorded have presented the principal forms symptomatic of hyperemia of the cerebral hemispheres. On combining with these few cases, which ended in death, several others of the same kind which terminated favourably, M. Andral lays it down, that cerebral congestion may present itself to us in one of the eight following forms.

The first form is characterized principally by dizziness of greater or less intensity: the patients may be affected at the same time with pain of head, dazzling, ringing in the ears, momentary aberrations of vision, temporary embarrassment in speeeh; a sense of formication in the limbs, and sometimes in the face. The countenance is in general flushed, eyes injected, pulse ordinarily not frequent, and of variable strengh. This state may last but for some moments, or some hours, but it may also be prolonged for several months, nay, continue even for several years. In some persons it shews itself but once; in others, it re-appears at intervals more or less remote. Our author has seen a man, 59 years of age, who, for the last 30 years, had not passed a single day without having, in different degrees, one or other of the symptoms abovementioned. Another person had experienced them since the age of thirty years till he was thirty-four. He then became completely freed from them till he was forty-eight years of age, when he was again attacked with violent dizziness. M. Andral has noticed the cases of several persons, in whom every year, nearly in the same month, these attacks of dizziness re-appeared. In some females, they manifest themselves regularly at the return of each menstrual period.

After this dizziness has lasted a shorter or longer time, it may happen that it attains all at once such an intensity, as to be changed into a sudden loss of consciousness; but the latter may likewise supervene without having been preceded by dizziness. It is this sudden loss of consciousness, with or without preceding dizziness, which characterizes the second form of cerebral congestion. In this form the patients fall to the ground, deprived suddenly of all intelligence, sensation, and motion; but, if their limbs be raised, they do not fall back again by their own weight, and some patients can sustain them in the air. There is not, then, properly speaking, any paralysis. They may remain in this state from some minutes up to twenty-four or thirty hours; then they come to themselves and are quickly restored, without retaining any lesion, either of sensation or motion. Others, after having come to themselves, retain for some days a little difficulty in the performance of some of the functions of the life of relation. Thus, their speech is embarrassed, or their different movements are difficult.

At the same time that the patients fall, deprived of consciousness, they may be struck with paralysis, either general, or confined to only one side of the body. This is the third form of cerebral congestion. But, almost at the same time that the loss of consciousness disappears, the paralysis is also seen to disappear, so that cerebral hemorrhage cannot be admitted to have taken place in this place. The cases above cited prove the possibility of this paralysis, without any effusion of blood having taken place into the brain. Instead of general or partial suspension of motion, this function may be performed in a manner disorderly and irregular, and without any participation of the will. Then, at the same time that there is loss of consciousness, there are observed either different convulsive movements, or permanent contraction of a certain number of muscles; all these symptoms last, at the utmost, for some hours—they then disappear, without leaving any trace behind. This constitutes the fourth form of cerebral congestion. In a fifth form, there is no longer loss of consciousness: it is paralysis that comes on at the very first, sometimes limited to certain muscles of the face, sometimes extended to the entire of one side of the body. This paralysis disappears very promptly, oftentimes a few hours after having commenced; and from this circumstance it is not to be presumed that it is connected with a hemorrhage, or softening. Our fourth case actually proves the contrary. The course of this paralysis was very remarkable in the following case. A middle-aged man, working in the quarries near Paris, was suddenly seized, on finishing his dinner, with a numbness of the right hand; an hour after, the entire upper extremity was totally deprived of motion; no pain was felt in it, nor did he complain of his head. At five o'clock in the evening, he had a sense of formication in the right foot; soon the power of motion was equally lost in the lower extremity of the right side: he entered the Hospital Cochin. On the following morning, the hemiplegia of the right side was complete; the sensibility of the paralysed limbs was still retained—he cannot move the right cheek, and, when he speaks, the left commissure of the lips is drawn up—the direction of the tongue is straight—intelligence perfect: he feels a numbness (this is his own expression) towards the frontal region—he was bled to sixteen ounces. In the course of the day, he was able to make some slight motion with the extremities of the right side. On the following morning, there was no trace of paralysis. This certainly is not the way in which the effects of cerebral hemorrhage disappear, or of any lesion affecting the interior of the nervous mass.

The sixth form of cerebral congestion is characterized by the sudden appearance of convulsive movements partial or general, without preceding loss of consciousness. These movements promptly disappear, without leaving any trace behind them. They may also come on, after the persons have experienced attacks of giddiness for a shorter or longer time, and the latter may even survive them. In a seventh form the cerebral congestion no longer produces coma; it no longer exercises any perceptible influence on the movements; the intelligence is the function here especially disturbed; violent delirium is observed, accompanied with great development of muscular strength, most frequently some time before death, the delirium is replaced by a state of coma, which becomes more and more profound: however, M. Andral states that he has seen cases in which up to the moment of death, the patients retained great agitation of the limbs, and ceased not to speak and vociferate. The most remarkable case of this kind which our author met was that of a middle-aged man, who for several hours uttered incessantly cries so loud as to disturb the rest of the entire ward. Suddenly he was no longer heard; when the attendant approached his bed, he was dead. A thunder-bolt could not have struck him more promptly. On opening the body no other lesion was detected except considerable injection of the nervous mass. He now notices the eighth form of cerebral congestion, in which continued fever appears at the commencement, at which time those symptoms principally predominate, which appertain to the first form of cerebral congestion already described. M. Andral particularly observed this form in some young soldiers, who were admitted in considerable numbers into the wards of the La Pitié, in the beginning of the Summer 1831. After laborious exercise several of these soldiers were seized with violent pains of head, vertigo, ringing of the ears; some even fell suddenly deprived of consciousness, and on coming to themselves they remained with the symptoms above detailed. On entering the wards a little time after the attack of their malady, they presented the following state: face red, eyes injected and moistened with tears; ringing of the ears, vertigo; great dizziness, which made them afraid of standing erect, lest they should fall. Frequent epistaxis; general debility—continual tendency to sleep; pulse strong and frequent; skin hot; no appreciable alteration with respect to the digestive or respiratory organs. This group of symptoms lasted from between three to twelve days; almost all of them were bled; some were merely subjected to the use of diluent drinks. By degrees the fever lessened, according as the symptoms of cerebral congestion disappeared. Though, as our author observes, it was not demonstrated that all the disease in these cases was seated in the brain, though there might have existed only mere general excitement, in which this organ participated, still the prevailing symptoms were always those of cerebral congestion; and on the removal of the fever, they were the only symptoms observed, and the only therapeutic indication was to combat them. Our author next considers those various causes under the influence of which cerebral congestions are found to occur. From his own experience, as well as from the accounts published by several medical men residing in different countries, he feels himself warranted in concluding, that these congestions find at least an occasional cause of development in the two extremes of temperature, and that they are reduced to their minimum of frequency under a mild and uniform temperature.

Baglivi in 1694, and Lancisi ten years after, saw apoplexy become so common in many parts of Italy, that they have actually described it as invested with all the characters of a genuine epidemic.

With respect to the influence of the quantity of electricity with which the atmosphere may be charged, on the production of cerebral congestion, our author professes himself unable to offer any opinion. He cites a fact however to prove that electricity, used as a therapeutic agent, may produce such a state. It was the case of a man, who had long been complaining of continual dizziness brought on by cerebral congestions, which were in general removed under the influence of bleeding and purging—he was at last attacked with apoplexy—loss of speech, irregular respiration, &c. By bleeding and purgation his condition was considerably ameliorated. After some months every encephalic symptom had disappeared—speech nearly restored; some degree of action had also returned to the affected limbs, when Dr. Strambio wished to try the effect of electro-puncture in order to bring back innervation to the semi-paralysed side. Dr. Fantonelli performed the operation as follows: he introduced a needle at the lower part of the neck on the side opposite the paralysed limbs, then another needle into the external malleolus of the affected leg; a metallic wire communicating with the two needles, was brought in contact with a voltaic pile of only five discs, so that the negative pole corresponded with the needle of the affected part: the introduction of the needles was not painful; but at each shock, acute pains and violent contractions were manifested in the muscles nearest to the needles, and particularly in those of the affected part. After five or six electric shocks they were obliged to desist, the pain becoming intolerable. The experiment was performed three times. After the first the patient was more cheerful, and his movements were more free; at the second he felt some uneasiness; at the third he was attacked with fever, and symptoms of cerebral congestion. Bleeding and revulsives quieted these symptoms very soon; but his former state returned. At present his speech is nearly gone, and the movements of his leg are very weak. Arnica and rhus radicans were tried but to no purpose, nay rather disadvantage. More positive researches than any yet made are necessary to establish how far a diet usually strong and exciting directly influences the production of cerebral congestion. In order that it should do so there must be at least a disposition on the part of the individual. Alcoholic liquors will, beyond all doubt, produce cerebral congestion. Nothing resembles some of the forms of cerebral congestion described more than intoxication. M. Andral states he had twice an opportunity of opening the bodies of persons, who, after indulging in strong liquors to excess, fell down drunk dead* (ivres morts) according to an expression consecrated by use. What he found was as follows: In both, the pia mater covering the convexity of the cerebral hemispheres was very much injected; the grey substance of the circumvolutions participated in this injection; the entire substance of the hemispheres was traversed by a great number of red points; the ventricles contained a moderate quantity of serum; the cerebellum was also injected, as well as its membranes, but not more than the brain. In no part was the consistence of the nervous mass altered. He found neither in the ventricles nor elsewhere any odour of alcohol, as was discovered within these ventricles in an individual whose case is given in Dr. Cooke's Work on Nervous Diseases. In this latter case the body was opened immediately after death; there was found in the ventricles a clear fluid which had the taste and smell of alcohol, and which took fire on being brought near a burning body. In one of the cases we examined, the mucous membrane of the stomach presented in several parts, nearly amounting to one third of the stomach, a surface dotted with bright red points; in the other, the gastric mucous membrane was of a white colour; it was not softened in either case.

Alcoholic liquors have not only caused cerebral congestions; they have also sometimes produced hemorrhage either around the brain into the subarachnoid cellular tissue, or into the nervous substance itself. These facts prove beyond a doubt that alcoholic liquors produce drunkenness by acting directly on the brain and not through the intervention of the stomach. Here is what was observed regarding the symptoms in one of the above-mentioned cases, (the second.) A man was brought to the La Charité about an hour after having drunk an enormous quantity of brandy and other alcoholic liquors. For the last half hour he had been in a state of the most profound coma; skin seemed insensible; respiration stertorous; pupils exceedingly dilated; pulse frequent and full. This state lasted without any

* The expression which use has consecrated among us, namely *dead drunk*, is used to signify not precisely the same thing as is here meant by *ivres morts*.—R.

change for twenty-four hours; it then ceased, and was suddenly replaced by furious delirium; the latter lasted about 15 hours; at the end of this time the coma returned; the respiration became more and more embarrassed, and the patient died. We have already seen the lesions found in the body. Active treatment was employed; he was twice bled; thirty leeches were applied to the neck; his head was covered with ice, and his lower extremities were surrounded with sinapisms. This group of symptoms, as well as the post mortem examinations, sufficiently prove the direct influence exercised on the brain by alcoholic preparations.

A great number of substances, ranked as narcotic poisons, have commonly the effect of determining in the brain a greater or less congestion. But certainly it is not by this congestion alone that the special phenomena produced by each of them can be explained. Let a man have been poisoned by alcohol, by opium, belladonna, tobacco, digitalis, camphor, prussic acid, &c. there will always be found in the brain, when examined after death, one of the same modifications, which will vary only in intensity; this will always be an hyperemia; and yet what can be more dissimilar than the functional disturbances, to which the use of these substances shall give rise. Beyond the hyperemia the only phenomenon which appears to us after death, there are then in the brain other modifications produced, which are no longer proved by the scalpel, but by the diversity in the nature of the symptoms observed during life. It is not then the cerebral congestion which is the cause of the specific symptoms which are produced by the different substances which have been just named; this congestion is but one of the elements of the morbid state to which they give rise, a secondary element, the intensity of which does not increase with the severity of the symptoms, and which may even be wanting, without the latter ceasing to exist. Is it true that the specific symptoms produced by each of them may be explained by the influence which each of them exercises over a particular part of the encephalon? Is it true, for instance, that opium acts especially on the cerebral hemispheres, alcohol on the cerebellum, belladonna on the tubercula quadrigemina? This is not the place to discuss the value of the physiological experiments by the aid of which an endeavour has been made to establish their specific actions. All that can be said is, that hitherto the observations made on man have not sufficiently demonstrated these results, neither have they disproved or invalidated them. However we shall remark that in these two cases of poisoning by alcohol which we have above mentioned, the congestion was seated in the cerebral hemispheres as well as in the cerebellum, and that the latter was not the seat of any specific lesion, at least of one which our present means of investigation will permit us to recognize. Besides, nothing is more variable, as every body knows, than the influence exercised on the encephalon by the different substances whose action we here examine. There are in this respect individual susceptibilities, instances of which have fallen under the observation of every medical man.

After having considered some of the external circumstances, which by the effects they produce on the animal economy may favour the development of cerebral hyperemia, he now directs his attention to the economy itself, and considers whether it will not present certain conditions, which may also have their share in the production of encephalic congestions. Among these conditions he reckons some states of the brain itself; thus forced exertion of the intellect is an unquestionable cause of cerebral congestion. He mentions the case of a young man, 27 years of age, who, after having devoted himself for a month unceasingly to very painful mental exertion, fell down suddenly, deprived of consciousness and motion: he was considered as struck with a severe attack of apoplexy; he was immediately bled: at the end of an hour he recovered the use of his senses; he was not paralysed: but his limbs on the right and left were as it were benumbed; he stammered, recovered not without difficulty the thread of his ideas, and stared at those around him with an astonished, and, as it were, a stupid air: these phenomena lasted for eight hours, continually diminishing; then they disappeared. There remained however a certain vagueness in his ideas, which did not leave him, till he remained for a considerable time in the country. Strong mental emotions have more than once produced cerebral congestions which have proved fatal.

Certain affections referrible to the brain may also determine in this organ a congestion, which medical men have sometimes erroneously taken for the cause of the disease, as epilepsy for example; but though not causing the attack itself, the congestion accompanying or succeeding it becomes itself the cause of certain morbid phenomena. It is on this that the cerebral phenomena seem to depend, which are often observed after the attack has terminated, as certain disturbances of the intellect, or else a state of coma, which lasts a longer or shorter time; or again, certain disturbances in the power of motion, as paralysis, or a momentary contraction of the limbs. The congestions which take place in the brain of epileptic patients, during the fit, leave also traces on their countenance. Thus several of them present, for the two or three days following the fit, slight ecchymoses on the skin of the cheeks and on the conjunctiva. Our author saw one instance in which after every fit, a broad livid spot, like that produced by a contusion, covered the forehead and eye-lids: this mark diminished gradually, and there was not a trace of it at the end of six or seven days.

Accidental products developed in the brain, old apoplectic cysts existing in this organ, must again be considered as so many thorns, which from time to time call around them, as around a centre of irritation, an hyperemia variable in intensity and extent; by the more or less frequent returns of this hyperemia are explained certain phenomena, intermitting as their cause, which appear at intervals in individuals labouring under a cerebral affection of long standing, phenomena most usually combated by bloodletting. In this way, in particular, may be explained those intermitting convulsions to which several children are subject, in whose brains tubercles exist; it often happens, that when once the convulsions have ceased, there no longer remains any cerebral symptom indicating the existence of the accidental product; a remarkable instance of intermitting phenomena produced by a constant lesion. The influence exercised by the different organs, in health or disease, on the production or return of cerebral congestions, merits particular attention. There is no doubt, for example, that in those who are predisposed, the process of digestion favours the return of these congestions; to a slight degree of these congestions, we may attribute the drowsiness exhibited by certain persons after meals. With respect to diseases of the stomach, they possess, in certain cases, a manifest influence on the development of cerebral congestions. Thus, at all ages, and particularly in infancy, acute gastro-enteritis may be accompanied by symptoms announcing the existence of an encephalic hyperemia. The same happens, though more rarely, in chronic gastro-enteritis.

The circulatory organs may, also, by the different states in which they may happen to be, produce different degrees of cerebral congestion. There cannot be a doubt but that the variable degrees of force with which the heart drives the blood towards the brain, may influence the production of cerebral hyperemia. Thus our author, and every medical man, may often see persons, in whom the increase of violence in the palpitations with which they were habitually attacked was constantly accompanied with vertigo, dizziness, ringing in the ears, and the other symptoms indicative of cerebral congestion; symptoms too often set down as purely nervous; some experience a sense of numbness at the ends of the fingers; all these phonomena cease, when the palpitations become less violent. Thus, our author feels himself warranted in stating, that increase in the force of the heart's impulse, whether it be nervous, or arising from hypertrophy of the organ, has an undoubted influence on the production of cerebral congestions.

It has been stated that an obstacle to the course of the arterial blood, below the arch of the aorta, must produce the same effect as hypertrophy of the left ventricle, and favour, in the same manner, the production of cerebral congestion. A case has even been published, in which an attack of apoplexy was considered owing to a tumor, which compressed the aorta a little below its passage through the diaphragm. If such a cause was real, it certainly should have its maximum of influence, as our author very properly observes, in cases where the aorta, immediately below its arch, was considerably contracted, or even obliterated: now, in the cases of this kind which have been recorded, there is no mention whatever, either of congestion or cerebral hemorrhage. When any obstacle whatever is opposed to the free return of blood from the brain to the heart, our author asks, Is the result of this a tendency to cerebral congestion? As a proof that such is the case, he refers to the sensation experienced by a person whose neck is very much squeezed; cerebral congestion has also been found in persons who have died by strangulation. Another question may be here started: should we, says M. Andral, rank among the causes productive occasionally of cerebral congestion the increase in the circulation, such as is produced by fever? Since, in this state, several of the tissues are reddened, is it not natural, he says, to suppose that the same will happen to the brain? There certainly is no other mode of accounting for the headache, vertigo, and other cerebral symptoms accompanying the access of fever. In the case of children, this morbid state is frequently accompanied with somnolence; we often see adults, also, very drowsy, when labouring under even a slight febrile attack; delirium too frequently is observed in such cases, which goes off according as the fever declines. The extreme sensibility to external impressions, the sense of fatigue, those dull pains in the limbs, and that feeling of general debility, which so often accompany well-marked fever; these surely are also phenomena which accompany certain forms of cerebral congestion. The real existence of the latter cannot be questioned in such cases: but here it must be observed that, far from being the cause of the febrile disturbance, the congestion is often but an effect of it. The production of cerebral congestions is also favoured by the inflammation of the different organs. The hyperemia, which does not constitute inflammation, but which is one of its elements, may be repeated on the brain, and this is observed, both when this inflammation still continues in all its force, and when it has prematurely disappeared. As an example of the first case, we shall instance erysipelas of the face or of the scalp, which sometimes terminates fatally by the cerebral symptoms which are complicated with it, and for the explanation of which no other lesion is found on examination, than a greater or less hyperemia of the encephalic mass. As an example of the second kind, we shall instance what occasionally happens during measles or scarlatina. In some children, the eruption has scarcely shewn itself when it begins to fade, and, at the same time, the face and eyes become very much injected; the children complain of headache; they become debilitated; all motion is painful to them ; they soon become comatose, and die. To account for these serious symptoms, what do we find in the encephalon ? Sometimes a sero-purulent infiltration of the meninges, or a perceptible distention of the ventricles by turbid or limpid serum ; but most frequently nothing more than simple hyperemia, which, in more than one case, is itself not very well marked.

It is, again, common enough to see signs of cerebral congestion come on during the febrile disturbance which precedes the eruption of small-pox, measles, or scarlatina; here we see many children attacked with convulsions, stupor, delirium, all which phenomena vanish when once the eruption comes out. Our author has hitherto considered the influence of the forces, whether mechanical or vital, which circulate the blood, in the production of cerebral congestion. He now asks—Are these congestions also influenced by the different degrees of activity of sanguification, that is, by the greater or less energy of the force which produces the blood ? In other words, what is the influence exercised either by a state of general plethora, or by a contrary state, on the production of cerebral hyperemia? Whilst he admits (an admission which cannot be refused by the way) that in many individuals a state of general plethora coincides with the appearance of the symptoms indicating the existence of cerebral congestion, he insists that it is far from being so in all cases, as there are, he says, individuals in whom this congestion appears at the very time when they were in a very obvious and perceptible state of anemia.

Our author next considers the very interesting subject of *masked intermitting fevers*, in which he details the case of a patient who exhibited all the phenomena of *intermittent apoplexy*, which in its returns observed the same regularity as the fevers of this name, and presented itself uniformly and regularly with precisely the same types. We observed some years ago, says our author, a very remarkable case of this kind, which we shall here record. A woman, 63 years of age, habitually enjoyed tolerably good health, when one morning on getting up, she was suddenly seized with great illness, vomiting and a violent pain of head; a quarter of an hour after these symptoms appeared, she uttered a loud scream, and fell, deprived of consciousness. We visit the patient about half an hour after the fall; we find her plunged into a profound coma ; eyes shut, pupils large and immoveable till the eye-lids are raised, and the conjunctiva is touched with the end of the finger, there is scarcely produced a slight contraction of the eye-lids, and the patient makes no effort to withdraw herself from this contact. Face injected; commissures of the lips not affected; tongue cannot be seen; the four extremities are in a state of complete relaxation, and the sensibility of the skin covering them appears abolished. The pulse strong and free from frequency—heart beats strongly. This woman appears to us struck with cerebral hæmorrhage considerable enough to engage both hemispheres; we immediately bleed her, and have her removed to the La Charité, after passing the most unfavourable prognosis on her state. What was our astonishment, when the next morning, at the visiting time, we found her sitting up in the bed, possessing all her intelligence, and enjoying all the freedom of motion. What occurred to her was as follows. After the bleeding no amendment manifested itself; she continued plunged in coma till towards six o'clock in the evening; then she came to herself, and, according to the account of the sister of the ward, she no longer appeared ill. From this we thought that the woman had nothing but a violent cerebral congestion, or what is called *coup de sang*.

The day passed on, and when we saw her the next day, she asked permission to leave the hospital; but before returning home, she was to pass through new sufferings. We had scarcely left her, (it was then 7 o'clock in the morning) when she was seized with vomiting, as on the day before, then she suddenly lost all sensation and motion; and the same symptoms which were observed on her before entering the hospital now returned. This time they lasted longer; in the evening they still continued; no amendment at night; and when we saw her again at 7 in the morning, she was still in a profound coma. However, she was bled, leeches were applied to her neck; up to one o'clock in the afternoon no change appeared; then the patient opened her eyes, spoke, possessed her intellects, moved her limbs with ease, and a second time was quite well. She then left the hospital. We visit her at her own house; she is quite well; yet she stammers a little; and there is a slight degree of stupor in her countenance. We do not yet suspect an intermitting apoplexy, and no particular medicinal directions were given. The following morning the same symptoms return. They continue the entire day with frightful intensity, and last 35 hours; then, as on the two preceding occasions, the patient comes to herself, and recovers the freedom of her motions. But her intellect is somewhat dull, and she speaks not without difficulty. We then asked ourselves whether we had not to do with one of those diseases described under the name of *masked intermittent fevers*. Ten or eleven hours still remained till the period when the next attack was to take place; we instantly administered by the mouth twenty grains of sulphate of quina; we administered the same dose of this salt in a starch injection, and we placed under each axilla, in each inguinal region, twelve grains of the same salt mixed up with some fresh butter. We await with anxiety for the patient, and also with ardent feelings of scientific curiosity for what will happen the following morning. Towards six o'clock no symptom developed itself; thus the fit is at least retarded, and there is reason to think that if it returns, it will be less intense. Towards noon the patient begins to feel a shivering, which she had not experienced on the preceding occasions; violent head-ache supervened without vomiting; soon after some convulsive movements agitate the muscles of the face; her intelligence becomes disturbed; movements of the limbs not yet changed; pulse accelerated; these phenomena succeed each other in the space of half an hour; then they are replaced by a state of coma which lasts for about two hours, and then goes off. The patient then continues for some time, as if benumbed; her skin becomes covered with a little moisture, and again she appears cured. Sulphate of quinine was immediately administered in the same dose, and after the same manner as on the day before; no symptoms of the complaint returned after.

This fact furnishes a very remarkable instance of an intermittent cerebral congestion, which assumed a tertian type, and which was completely stopped by quinquina. It was observed that the third fit was more severe than the preceding two. The sulphate of quinine was administered, and the next fit becomes less severe and of less duration, and it is also very much modified as to its symptoms; now for the first time some shivering marked its onset, and for the first time also its termination is accompanied with some perspirations. So that in becoming less severe, the fit approaches in its symptoms a fit of ordinary intermitting fever.

We have now closely followed M. Andral in his detail of the causes, which usually determine cerebral congestions; and have been somewhat surprised, nay perhaps disappointed, that among them we find no mention made, at least with that explicitness it deserves, of certain other circumstances, which are so frequently found to determine this phenomenon, we mean, the different pathological states of the lungs; such as condensation from the presence of effused fluid in the pleura; changes in the bronchial mucous membrane from chronic inflammation—emphysema of the lungs—we know that many of the most distressing symptoms of bronchitis, as Dr. Bright well remarks, namely the intense headache, the wandering delirium, and the lethargic coma so often observed in bronchitic patients, are indisputably dependent on the state of the circulation through the brain. It is not however quite evident, how far the mere mechanical congestion may operate in producing these symptoms; the chemical condition of the blood must also be taken into account in the estimate.

Our author having sketched the principal traits of the history of cerebral congestions, starts the following questions; Are the symptoms characterising the different forms of cerebral congestions connected in all cases with the afflux of a too great quantity of blood towards the brain? Do they depend solely on this cause? Are they not sometimes seen as the effect of a quite opposite state of the nervous centres, or in other words, as the effect of their anemia? It is, he says, and we fully agree with him, a pathological law that, in every organ, the diminution of its normal quantity of blood will produce functional disturbances in it, as well as a hyperemic state of it. Nay what is more, these functional disturbances are at times precisely similar. Thus, let an impoverished thin blood circulate through the heart, and palpitations of this organ will be the result, just as if it contained too much blood. Dyspnœa will come on equally, whether the lung be the seat of greater or less hyperemia, or whether the air, on entering the pulmonary vesicles, no longer finds sufficient blood to act upon. Our author has frequently seen patients, who appeared completely anemic; their face quite pale, and their skin presenting a colour like that of wax; the least exertion set them panting for breath—they were subject to violent palpitations of the heart, painful and laborious digestion. These persons at the same time complained of head-ache, dizziness, vertigo, tinnitus aurium; some even occasionally, or even continually, had numbness of the limbs—others were annoyed with hallucinations in sight and hearing. These individuals had been for a long time subject to copious hæmorrhages by different outlets, either through the nasal fossæ, the rectum, or uterus. In all such cases it must be admitted that the brain is disturbed in its functions, in consequence of being no longer stimulated or nourished by the blood sent into it; which blood has now become too poor in quality, or too small in quantity.

If now, observes our author, we leave the mere observation of facts, and endeavour to account for them, we shall soon be convinced of the insufficiency of the Brunonian theory to explain the very frequently similar symptoms, which result from cerebral hyperemia and cerebral anemia. These symptoms do not necessarily indicate either a hypersthenic, or an asthenic state. This may be owing to a mere perversion of the cerebral influence, a perversion not to be referred to a plus or a minus of vitality, but resulting from the circumstance of the brain's no longer receiving its normal quantity of blood, and not merely because it is then less excited.

But this is not all; when we have referred the symptoms to hyperemia in one case, and to anemia in another, have we come to the bottom of the matter? By no means; for this hyperemia and this anemia are themselves but effects, which may be often produced by the same influence: thus, by a mental emotion, the skin of the face becomes red in one person, and pale in another.

In the nervous centres, as in other parts, before the production of hyperemia or anemia, we must conceive a primary modification of that force, whatever it be, which subjects the cerebral circulation to certain rules. Amidst these numerous currents, these oscillations of globules, which are going on within the organic tissues, how many causes always present, whose influence is wholly unknown to us, may derange a current and modify the distribution of the globules. On attentively considering the merits of this question we shall soon arrive at the conclusion that hyperemia, and anemia, in the brain, are themselves but secondary phenomena, mere effects. But these effects, inconstant and variable, do not necessarily follow the action of the cause; they may be wanting, and yet the symptoms will still remain! For they depend less on the state of cerebral hyperemia and anemia, than on the organic modification, which precedes and produces them.

Having followed our author thus far in his cases of and observations on cerebral congestion, we shall in our next Number present our readers with the subject of hæmorrhage of the cerebral hemispheres.

